# Overcoming inefficient cellobiose fermentation by cellobiose phosphorylase in the presence of xylose

**DOI:** 10.1186/1754-6834-7-85

**Published:** 2014-06-07

**Authors:** Kulika Chomvong, Vesna Kordić, Xin Li, Stefan Bauer, Abigail E Gillespie, Suk-Jin Ha, Eun Joong Oh, Jonathan M Galazka, Yong-Su Jin, Jamie H D Cate

**Affiliations:** 1 Department of Plant and Microbial Biology, University of California, Berkeley, CA, USA; 2 Department of Chemistry, University of California, Berkeley, CA, USA; 3 Department of Molecular and Cell Biology, University of California, Berkeley, CA, USA; 4 Energy Biosciences Institute, University of California, Berkeley, CA, USA; 5 Department of Food Science and Human Nutrition, University of Illinois, Urbana, IL, USA; 6 Institute for Genomic Biology, University of Illinois, Urbana, IL, USA; 7 Department of Bioengineering and Technology, Kangwon National University, Chuncheon, Republic of Korea; 8 Physical Biosciences Division, Lawrence Berkeley National Laboratory, Berkeley, CA, USA

**Keywords:** Cellobiose, Cellobiose phosphorylase, Glucopyranosyl-xylose, Inhibition, Xylose

## Abstract

**Background:**

Cellobiose and xylose co-fermentation holds promise for efficiently producing biofuels from plant biomass. Cellobiose phosphorylase (CBP), an intracellular enzyme generally found in anaerobic bacteria, cleaves cellobiose to glucose and glucose-1-phosphate, providing energetic advantages under the anaerobic conditions required for large-scale biofuel production. However, the efficiency of CBP to cleave cellobiose in the presence of xylose is unknown. This study investigated the effect of xylose on anaerobic CBP-mediated cellobiose fermentation by *Saccharomyces cerevisiae*.

**Results:**

Yeast capable of fermenting cellobiose by the CBP pathway consumed cellobiose and produced ethanol at rates 61% and 42% slower, respectively, in the presence of xylose than in its absence. The system generated significant amounts of the byproduct 4-O-*β*-d-glucopyranosyl-d-xylose (GX), produced by CBP from glucose-1-phosphate and xylose. *In vitro* competition assays identified xylose as a mixed-inhibitor for cellobiose phosphorylase activity. The negative effects of xylose were effectively relieved by efficient cellobiose and xylose co-utilization. GX was also shown to be a substrate for cleavage by an intracellular β-glucosidase.

**Conclusions:**

Xylose exerted negative impacts on CBP-mediated cellobiose fermentation by acting as a substrate for GX byproduct formation and a mixed-inhibitor for cellobiose phosphorylase activity. Future efforts will require efficient xylose utilization, GX cleavage by a β-glucosidase, and/or a CBP with improved substrate specificity to overcome the negative impacts of xylose on CBP in cellobiose and xylose co-fermentation.

## Background

Cellulosic biofuels could make significant contributions to meet the ever-rising demand for energy. To economically produce fuels from cellulosic biomass, sugars derived from cellulose as well as hemicellulose must be utilized completely [1-3]. The co-fermentation of cellobiose derived from cellulose and xylose derived from hemicellulose allows these sugars to be consumed simultaneously [4], and may enable continuous biofuel production [5]. In this system, cellobiose and xylose are transported into engineered *S. cerevisiae* using a cellodextrin transporter (that is, CDT-1 from *Neurospora crassa*) and endogenous hexose transporters, respectively [4,6]. Intracellular cellobiose is then hydrolyzed into two molecules of glucose by an intracellular β-glucosidase (NCU00130; GH1-1) [6]. At the same time, xylose is consumed by an oxidoreductive pathway, comprising xylose reductase and xylitol dehydrogenase, that converts xylose to xylulose [7,8]. Alternatively, xylose can be utilized by xylose isomerase, which converts xylose directly to xylulose [9]. Glucose and xylulose are then metabolized using glycolysis and the pentose phosphate pathway, respectively, resulting in ethanol production [4].

A pathway potentially better suited to the anaerobic environment of large-scale biofuels production substitutes cellobiose phosphorolysis for the hydrolytic reaction of β-glucosidase [10,11]. This pathway comprises cellobiose phosphorylase (CBP), which cleaves intracellular cellobiose into glucose and glucose-1-phosphate (G1P) [12]. The phosphorolytic pathway requires one less ATP for each molecule of cellobiose to be metabolized by glycolysis. This is because glucose generated by hydrolysis of cellobiose requires two ATP molecules for hexokinase generation of glucose-6-phosphate (G6P) [13], whereas CBP uses inorganic phosphate in place of one of the ATP molecules to produce G1P. G1P can then be converted to G6P without the need for ATP by the enzyme phosphoglucomutase [14]. Under anaerobic conditions, in which glycolysis generates only two ATP molecules per glucose, increased ATP can result in increased biomass at the expense of ethanol product yield [15]. However, the phosphorolytic pathway can be engineered to perform better than the hydrolytic pathway in terms of product yield in stressful conditions like those expected in lignocellulosic hydrolysates [10].

Although the cellobiose phosphorolytic pathway has potential advantages, its efficiency, hereafter defined as incomplete or low rate of consumption, in the context of cellobiose and xylose co-fermentation is not known. Previously, cellobiose phosphorolytic pathways were combined with xylose isomerase pathways to construct anaerobic cellobiose and xylose co-fermentation systems in *Saccharomyces cerevisiae* and in *Escherichia coli*[16,17]. However, they are inefficient in terms of sugar consumption and ethanol production rates [16], or the systems remain to be fully optimized [17]. CBP from *Ruminococcus albus* NE1 (RaCBP) uses xylose as a substrate for the reverse of the phosphorolytic reaction [18]. We therefore hypothesized that the inefficiency in cellobiose and xylose co-fermentation previously observed was due to xylose interference with cellobiose consumption via CBP. The presence of xylose is unavoidable because it is a major component of hemicellulose, which has to be utilized for economical biofuel production [1-3]. We therefore tested the effect of xylose on CBP cellobiose fermentation, as well as two potential approaches to alleviate inefficient CBP-mediated cellobiose fermentation in the presence of xylose.

## Results

### Inefficient cellobiose fermentation in the presence of xylose

**Figure 1:**
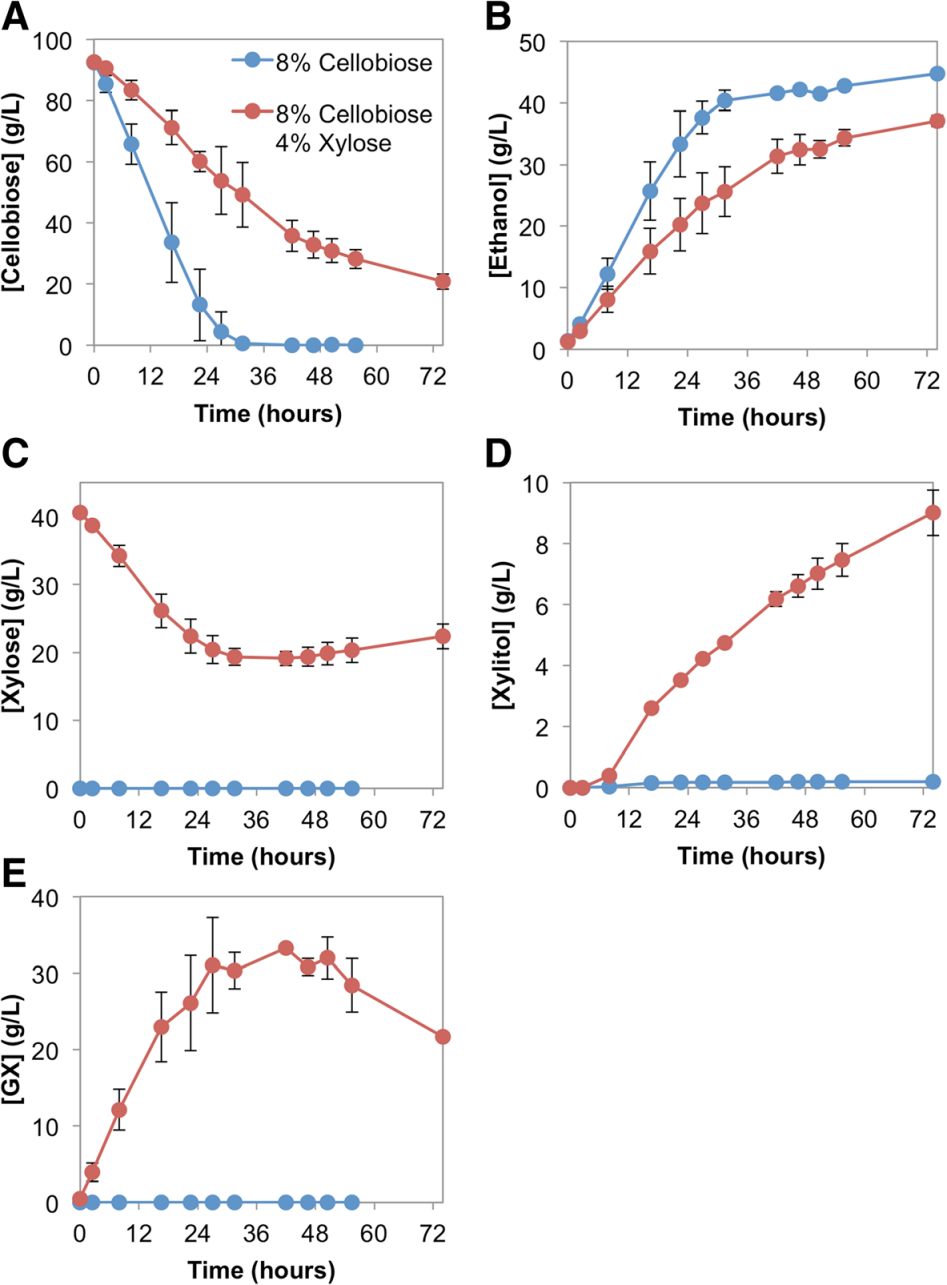
**Fermentation profile of engineered strain D452-2 in the presence and absence of xylose.***S. cerevisiae* strain D452-2 was transformed with the pCS plasmid, encoding cellodextrin transporter *cdt1*-F213L and SdCBP. Anaerobic fermentations were supplied with 80 g/L cellobiose in the presence (red dots) and absence (blue dots) of 40 g/L xylose. Extracellular concentrations of **(A)** cellobiose, **(B)** ethanol, **(C)** xylose, **(D)** xylitol and **(E)** glucopyranosyl-xylose are shown. Values and error bars represent the means and standard deviations of two independent biological replicates.

A codon-optimized CBP gene from *Saccharophagus degradans* (SdCBP) [10] and a mutant cellodextrin transporter encoding *N. crassa* CDT-1 (F213L) [10] were cloned into the 2μ plasmid pRS426 under the control of constitutive P_*PGK1*_ promoters (hereafter called pCS plasmid). *S. cerevisiae* strain D452-2 transformed with this plasmid was used for anaerobic fermentations (Table 1). The fermentations were carried out with 80 g/L of cellobiose as a carbon source, either with or without 40 g/L of xylose present. The engineered strain was capable of fermenting cellobiose to ethanol in both conditions (Figure 1A,B). However, in the presence of xylose, the rates of cellobiose consumption and ethanol production decreased by 61% and 42%, respectively (Figure 1A,B, Table 2). As a result, approximately 20 g/L of cellobiose remained in the fermentation broth after 72 hours in the presence of xylose (Figure 1A), whereas all of the cellobiose was consumed within 36 hours in the absence of xylose (Figure 1A). These results indicated that the presence of xylose had a severely negative impact on cellobiose fermentation mediated by CBP.Interestingly, in the fermentation supplied with 40 g/L of xylose, the xylose concentration showed an initial decrease followed by a slight recovery after 36 hours (Figure 1C). Xylitol was also produced with a titer of approximately 9 g/L at 72 hours (Figure 1D). These results suggest that approximately half of the xylose transported into the cell was reduced to xylitol but the rest remained unaccounted for.

**Table 1:** Strains and plasmids used in this study

**Strains/plasmids**	**Characteristics**	**Reference**
**D452-2**	*MATalpha, leu2, his3, ura3, and can1*	[19]
**SR8**	*ald6Δ* of the evolved strain D452-2 *leu2*::*LEU2* P_*TDH3*_-*XYL1*-T_*TDH3*_; *ura3::URA3* P_*TDH3*_*-XYL1-*T_*TDH3*_ P_*PGK1*_*-XYL2-*T_*PGK1*_ P_*TDH3*_*-XYL3-*T_*TDH3*_; *his1::HIS1* P_*PGK1*_*-XYL2-*T_*PGK1*_ P_*TDH3*_*-XYL3-*T_*TDH3*_	[20]
**SR8-a**	SR8 *ura3*	Courtesy of Dr. Soo Rin Kim
**pCT**	pRS426- P_*PGK1*_-*cdt*-1(F213L)-eGFP-t_*CYC1*_	[10]
**pSd**	pRS425- P_*PGK1*_-SdCBP-t_*CYC1*_	[10]
**pCS**	pRS426-P_*PGK1*_-*cdt-1*(F213L)-eGFP-t_*CYC1*_-P_*PGK1*_-SdCBP-t_*CYC1*_	This study
**pET-Sd**	pET302-NT/6his-SdCBP	This study

**Table 2:** Fermentation parameters

**Strain**	**Media**	**Cellobiose consumption rate (g/L · h)**	**Ethanol production rate (g/L · h)**
D452-2	Cellobiose	3.6 ± 0.05	1.5 ± 0.03
D452-2	Cellobiose + xylose	1.4 ± 0.04	0.85 ± 0.02
SR8-a	Cellobiose	3.7 ± 0.09	1.5 ± 0.04
SR8-a	Cellobiose + xylose	2.9 ± 0.08	1.6 ± 0.04

### *In vitro* and *in vivo* production of glucopyranosyl-xylose

**Figure 2:**
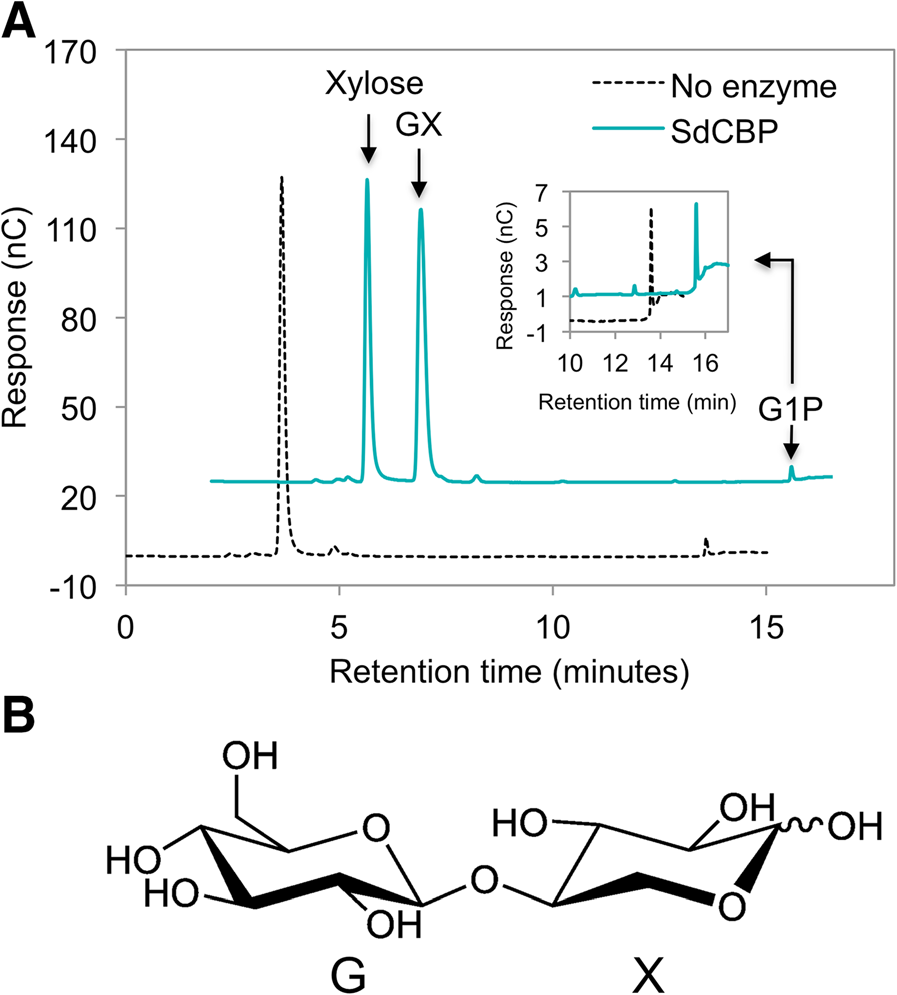
***In vitro *synthesis of glucopyranosyl-xylose catalyzed by purified *Saccharophagus degradans*cellobiose phosphorylase. (A)** Reactions containing 10 mM xylose and 10 mM G1P, along with 20 nM purified SdCBP in 20 mM 2-(N-morpholino) ethanesulfonic acid, pH 6.0 solution were incubated at 37°C for 12 hours. The purified SdCBP was omitted from the negative control. A product signal (eluted at 4.9 minutes), along with decreases in xylose and G1P (showed in the insert with different scale) signals, were observed only when SdCBP was provided in the reaction. Chromatograms are displayed with 2 minute-offset between samples. **(B)** Structure of the GX dimer. G1P, glucose-1-phosphate; GX, 4-O-β-D-glucopyranosyl-D-xylose; SdCBP, *Saccharophagus degradans* cellobiose phosphorylase.

To determine the fate of xylose that was unaccounted for by xylitol production, xylose and G1P were used as substrates for the reverse reaction catalyzed by purified SdCBP. Chromatograms of the reaction analyzed by ion chromatography showed a decrease in xylose and G1P concentration along with the appearance of a new peak (Figure 2A). Analysis of the reaction by mass spectrometry (MS) identified the molecular mass of the product to be 312 g/mol, consistent with that of 4-O-*β*-D-glucopyranosyl-D-xylose (GX) (Figure 2B, Additional file 1: Figure S1A). Tandem mass spectrometry (MS-MS) further indicated that the product comprised one hexose unit and one pentose unit (Additional file 1: Figure S1B). These results suggested that the *in vitro* reverse reaction of SdCBP produced a GX dimer when xylose and G1P were provided as substrates.

Using GX synthesized *in vitro* as a standard, GX was detected in the fermentation broth when cellobiose and xylose were supplied to yeast engineered with the CBP cellobiose consumption pathway (Figure 1E). Interestingly, the concentration of GX initially increased but started to decrease after 36 hours, with its highest concentration reaching approximately 30 g/L (Figure 1E). Extracellular concentrations of xylitol and GX combined accounted for 88% to 100% of the imported xylose (Additional file 1: Figure S2). Thus, yeast utilizing cellobiose by the CBP consumption pathway formed GX from intracellular xylose and G1P, in addition to converting some of the imported xylose to xylitol.

### Competition assays identified xylose as a mixed-inhibitor

**Figure 3:**
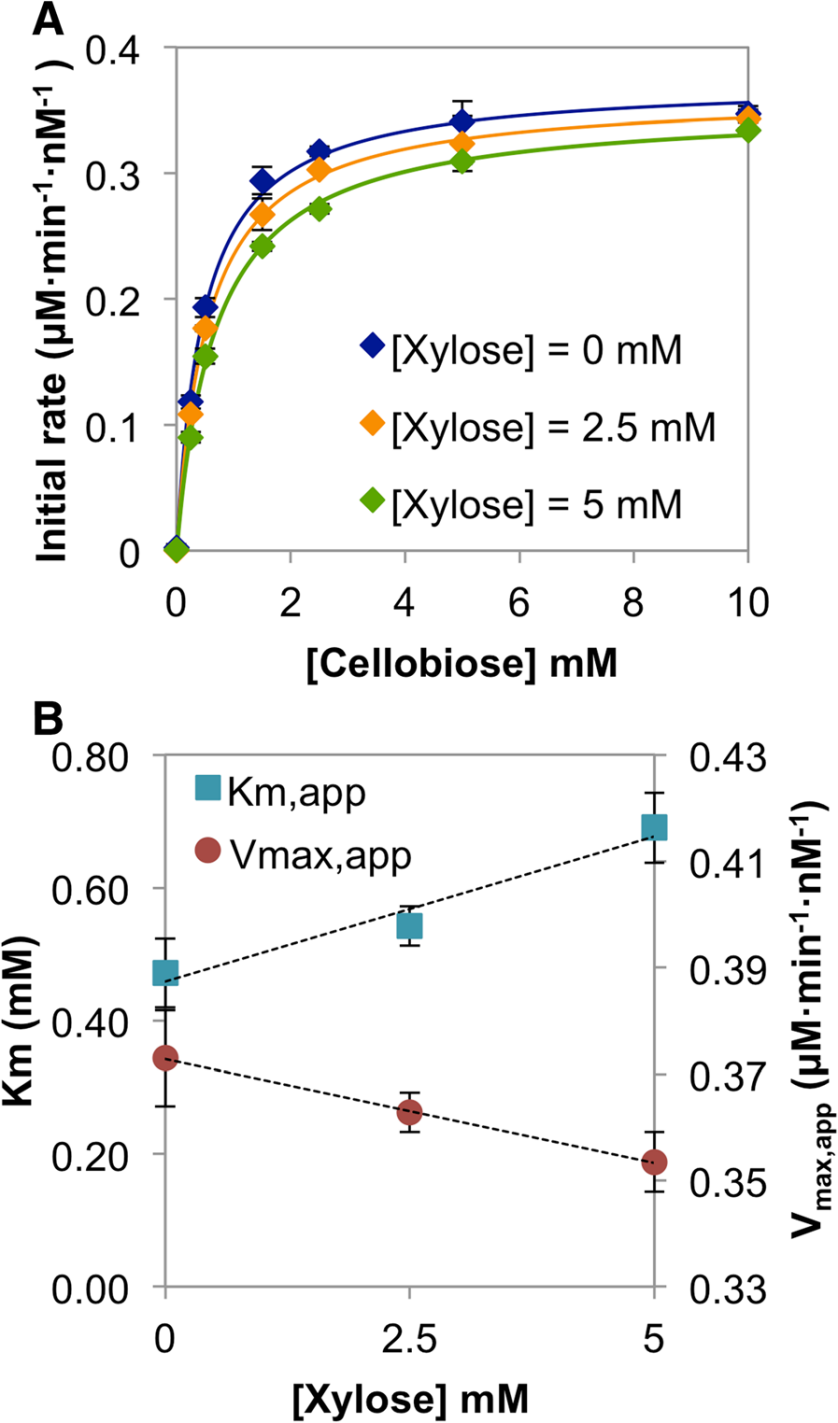
**Xylose competition assay of *Saccharophagus degradans *cellobiose phosphorylase activity.** The catalytic properties of SdCBP were determined in the presence of 0, 2.5 and 5 mM of xylose. **(A)** Initial rates of cellobiose phosphorolysis were calculated from the amount of continuous G1P production at different cellobiose concentrations. All reactions were carried out in duplicate. **(B)** Apparent kinetic parameters of SdCBP supplemented with 0, 2.5 and 5 mM of xylose determined by non-linear curve fitting.

To investigate the inhibitory effect of xylose on SdCBP activity, the catalytic properties of SdCBP were determined in the presence of varying xylose concentrations (Figure 3), at the time points preceding the production of GX (Additional file 1: Figure S3). Initial rates of cellobiose phosphorolysis were calculated from the amount of G1P produced at different cellobiose concentrations (Figure 3A). As the concentration of xylose increased, the apparent maximal rate (V_max,app_) linearly decreased while the apparent substrate concentration at which the reaction rate is half of V_max,app_ (K_M,app_) linearly increased (Figure 3A,B). This result indicated that the apparent affinity of CBP for cellobiose and its maximal phosphorolytic rate for cellobiose were inversely proportional to the xylose concentration, identifying xylose as a mixed-inhibitor for the cellobiose phosphorolysis reaction (Figure 3B). The negative impact of xylose on SdCBP phosphorolytic activity may therefore contribute to the decrease in cellobiose consumption rate observed phenotypically in the above fermentations (Figure 1A), in addition to the production of GX.

### Reduced glucopyranosyl-xylose formation in an efficient xylose utilizing strain, SR8-a

**Figure 4:**
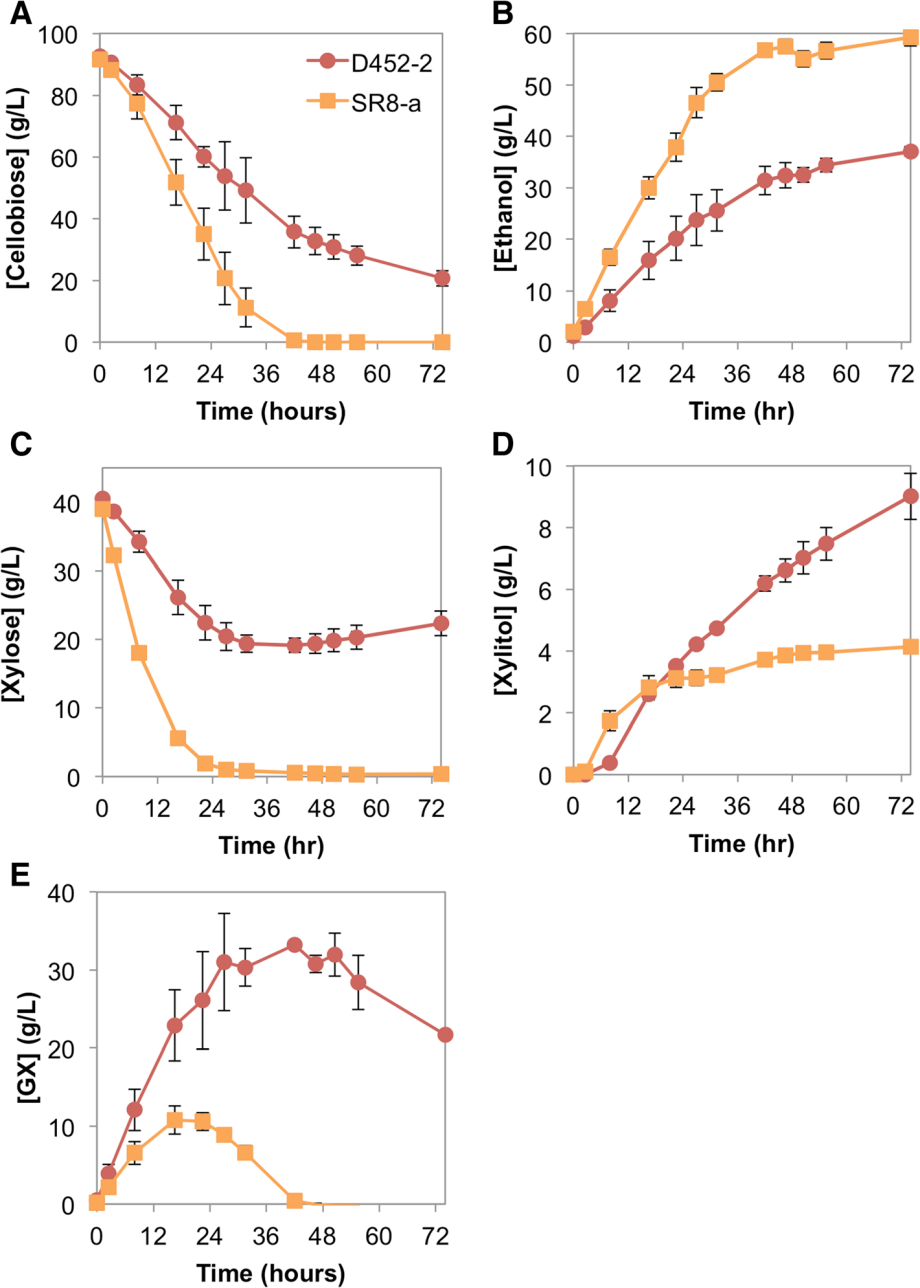
**Fermentation profile of engineered D452-2 and SR8-a strains supplemented with cellobiose and xylose. ***S. cerevisiae* D452-2 (red circle) and SR8-a (orange square) strains were transformed with the pCS plasmid. Anaerobic fermentations were supplied with 80 g/L cellobiose and 40 g/L xylose. Extracellular concentrations of **(A)** cellobiose, **(B)** ethanol, **(C)** xylose, **(D)** xylitol and **(E)** glucopyranosyl-xylose are shown. Values and error bars represent the means and standard deviations of two independent biological replicates.

Strain SR8-a is an engineered *S. cerevisiae* strain capable of rapid xylose fermentation [20]. We wondered whether rapid xylose utilization, which would maintain a lower intracellular concentration of xylose, could mitigate formation of GX by CBP. Strain SR8-a (Table 1) and strain D452-2 used above, which lacks a xylose-utilization pathway, were transformed with the pCS plasmid and fermentations were carried out with 80 g/L of cellobiose as a carbon source, with or without 40 g/L of xylose present. In the absence of xylose, the cellobiose consumption and ethanol production profiles of the engineered D452-2 and SR8-a strains were equivalent (Additional file 1: Figure S4). Notably, in the presence of xylose, the cellobiose consumption rate of the SR8-a strain was two-fold higher than that of the D452-2 strain (Figure 4A, Table 2). The SR8-a strain completely consumed cellobiose in 40 hours and xylose in 24 hours (Figure 4A,C). In the SR8-a background, GX was produced at a lower concentration, at a slower rate, and started to decrease after 16 hours, in comparison to 48 hours in the D452-2 strain (Figure 4E). In addition, the engineered SR8-a strain produced less xylitol and more ethanol than the D452-2 strain (Figure 4B,D). This result suggested that efficient xylose utilization reduced the formation of GX and increased the CBP-mediated cellobiose consumption rate in the presence of xylose.

The cellobiose consumption rate of the SR8-a strain supplemented with xylose was not as rapid as that of the D452-2 strain with no xylose present, showing a 22% decrease (Figure 1A, Figure 4A, Table 2). However, the negative effect of xylose on cellobiose consumption was alleviated in comparison to the 61% decrease in cellobiose consumption rate with the D452-2 strain in the presence of xylose (Figure 1A).

### Cleavage of glucopyranosyl-xylose by intracellular β-glucosidase and β-xylosidase

**Figure 5:**
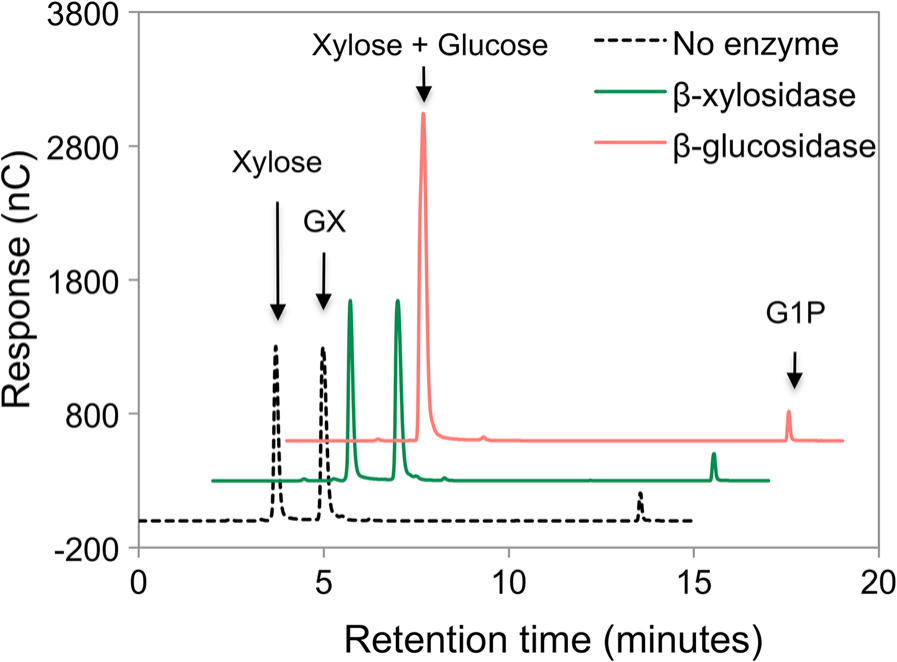
**β-xylosidase (NCU01900) and β-glucosidase (NCU00130) activities on glucopyranosyl-xylose.** GX synthesized *in vitro* used as a substrate contained some traces of xylose and G1P. Purified β-xylosidase and β-glucosidase were incubated with GX substrate mixture in 1× PBS, pH 7.4 buffer. The purified proteins were omitted in the control reaction. Chromatograms are displayed with 2 minute-offsets between samples. GX, glucopyranosyl-xylose; G1P, glucose-1-phosphate.

Given that the GX formed by CBP in the reverse phosphorolytic reaction should have a β-1,4 linkage, we wondered whether GX might serve as a substrate for either an intracellular β-xylosidase (NCU01900) or the intracellular β-glucosidase (NCU00130; GH1-1) from *N. crassa*. Although the amount of GX did not change significantly when the β-xylosidase or no enzyme was used (Figure 5), the β-glucosidase GH1-1 completely hydrolyzed GX, as indicated by the disappearance of the GX peak (Figure 5). Signals of the hydrolysis products, namely glucose and xylose, overlapped but the increase was detected (Figure 5). Further enzymatic analysis of β-glucosidase GH1-1 activity revealed that its maximal rate for GX cleavage was three times lower and its K_M_ for GX was seven-fold higher than that for cellobiose (Table 3, Additional file 1: Figure S5). These results showed that the β-glucosidase GH1-1, but not the β-xylosidase NCU01900, was capable of cleaving GX to glucose and xylose. However, cellobiose may compete with GX hydrolysis in the context of *in vivo* cellobiose consumption.

**Table 3:** Kinetic parameters of β-glucosidase GH1-1 for glucopyranosyl-xylose and cellobiose

**Kinetic parameters**	**Glucopyranosyl-xylose**	**Cellobiose**
K_M_ (mM)	3.5 ± 1.4	0.49 ± 0.05
V_max_ (μM · min^-1^ · nM^-1^)	0.48 ± 0.10	1.3 ± 0.04

## Discussion

In this study, we identified GX production as a competing off-pathway product of cellobiose and xylose co-fermentation when cellobiose is consumed using a CBP-mediated pathway. The production of GX results in a decreased efficiency of cellobiose fermentation (Figure 1), especially in the absence of a xylose-consumption pathway. The decrease in extracellular xylose concentration (Figure 1C) indicates that xylose is transported into the cell, likely via endogenous hexose transporters. Though lacking a xylose utilization pathway, the engineered yeast strain D452-2 expressing the pCS plasmid is expected to convert some xylose to xylitol by means of endogenous aldose reductase (Gre3) activity [21], consistent with the fact that xylitol was detected in the fermentation medium (Figure 1D). We further verified that much of the remaining xylose loss in the intracellular pool was due to its conversion to GX by the reverse phosphorolysis reaction catalyzed by CBP between G1P and xylose (Additional file 1: Figure S2). Interestingly, we found that the extracellular concentration of GX eventually starts to decrease, and at the same time that of xylose begins to recover (Figure 1C,E).

**Figure 6:**
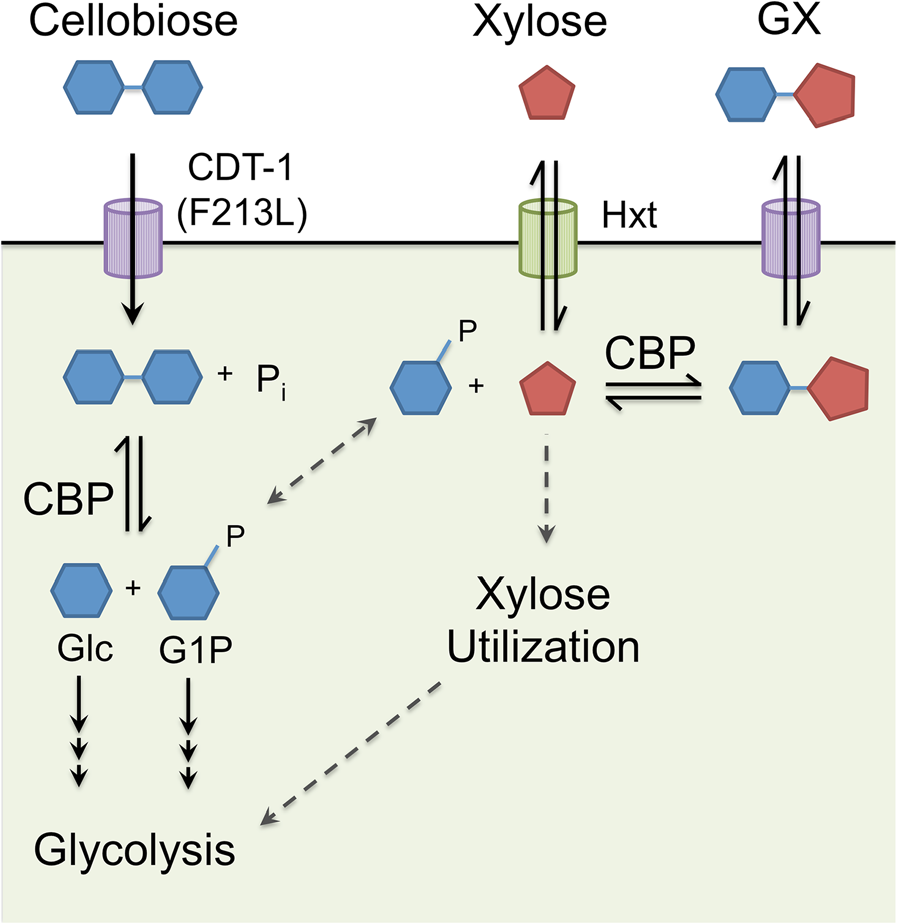
**Model of cellobiose phosphorylase-mediated cellobiose consumption in the presence of xylose.** Cellobiose and xylose are simultaneously imported via the cellodextrin transporter CDT-1 (F213L) and endogenous hexose transporters, respectively. Cellobiose undergoes phosphorolytic cleavage via CBP, generating glucose and G1P, both of which enter glycolysis. However, some of the G1P and imported xylose are condensed by CBP to produce GX in its thermodynamically favorable reverse reaction. GX is then transported out of the cell and imported back into the cell by the cellodextrin transporter over the time course of fermentations. The intracellular GX is then cleaved to G1P and xylose by CBP when the intracellular cellobiose concentration drops in later times of the fermentation. Free xylose is then released back into the fermentation broth in the absence of the xylose consumption pathway. CDT-1 (F213L), cellodextrin transporter mutant; Hxt, hexose transporters; CBP, cellobiose phosphorylase; P_i_, inorganic phosphate; Glc, glucose; GX, glucopyranosyl-xylose; G1P, glucose-1-phosphate.

We propose that, in the presence of xylose, cellobiose is first transported into yeast cells via cellodextrin transporter mutant CDT-1 (F213L) and cleaved by CBP to generate glucose and G1P (Figure 6). At the same time, xylose is transported into the cell via hexose transporters. With xylose and G1P present inside the cell, CBP catalyzes the reverse phosphorolysis reaction, producing GX, which can be exported by the cellodextrin transporter (Figure 6). The model explains the initial decrease in xylose concentration and increase in GX concentration in the media (Figure 1C,E). CDT-1, a proton symporter [22], can reversibly transport substrates [4] because of the thermodynamic driving forces of high intracellular substrate (that is, GX) concentrations competing with high extracellular proton concentrations. At later stages of the fermentation, GX in the media is transported back into the cell via the cellodextrin transporter, along with the rest of the cellobiose (Figure 6). GX is then cleaved by SdCBP to generate G1P and xylose. Some xylose released is then exported back into the media via endogenous hexose transporters. The second part of the model explains the decrease in GX concentration and the increase in xylose concentration in the media (Figure 1C,E).Formation and cleavage of GX as observed here is unfavorable to cellobiose fermentation. GX exhausts resources that could have been dedicated to cellobiose consumption, namely the use of CDT-1 to export and import GX and SdCBP to form and cleave GX. This is especially deleterious because GX processes occur simultaneously with cellobiose consumption (Figures 1A,E and 6). Furthermore, GX formation requires G1P as one of the substrates (Figure 2A). G1P is thus diverted from glycolysis, where in the absence of xylose it would be converted to G6P by phosphoglucomutase. GX formation would thereby decrease the rate of ethanol production because some of G1P produced from cellobiose via CBP is wasted in the formation of GX. These considerations suggest that GX formation likely results in a decrease in cellobiose import by CDT-1, slows cleavage of cellobiose, and reduces the rate of ethanol production. Extracellular concentrations of cellobiose and ethanol also support this model (Figure 1A,B).

The reported catalytic efficiency (k_cat,app_/K_M,app_) of RaCBP for xylose in the reverse phosphorolysis reaction is only 1% of that for glucose [18]. However, with SdCBP, a substantial amount of GX formation is observed (Figure 1E). This may be explained by differences between the two CBPs, or by the high concentrations of intracellular xylose and low concentrations of intracellular glucose present in our experiments. When the xylose utilization pathway is absent, high intracellular xylose concentrations are expected. Although we did not measure the intracellular concentration of xylose directly, it is expected to be similar to the extracellular concentration due to the fact that xylose is imported by hexose transporters, which are facilitators [23,24]. Thus, the imported xylose not accounted for by xylitol production (Figure 1C,D), would result in an intracellular concentration of xylose near or above the reported K_M,app_ of RaCBP for xylose (25 mM) [18]. By contrast, the intracellular concentration of glucose is expected to be small because glucose can be efficiently converted to G6P and consumed by glycolysis. The maximal reported free intracellular glucose concentration is 2 to 3 mM [25], slightly above the K_M,app_ of RaCBP on glucose (1.5 mM) [18]. Furthermore, the CBP reverse reaction is thermodynamically favorable, with a ΔG° = -3.6 kJ mol^-1^ for cellobiose formation [10,12]. Thus, the amount of GX formation we observed was consistent with the known thermodynamic and kinetic properties of the enzymes used in the CBP-mediated cellobiose consumption pathway, especially due to the drive from a high intracellular xylose concentration.

**Figure 7:**
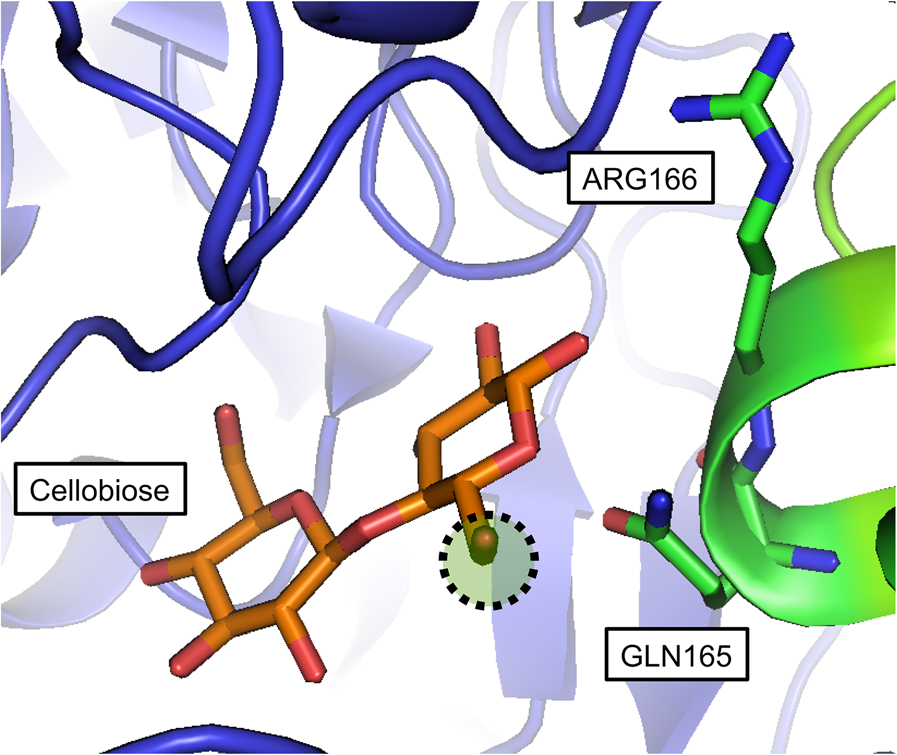
**Active site of *Cellulomonas uda *cellobiose phosphorylase in complex with cellobiose.** The crystal structure of *C. uda* CBP is shown in complex with cellobiose [PDB:3S4A]. CBP is a homodimer whose active sites comprise an (α/α)_6_-barrel domain of one subunit (blue) and the helical extension from the N-terminal domain of the adjacent subunit (green). Cellobiose is bound in the active site with its reducing end pointing toward the N-terminal extension from the adjacent subunit (green). Arg166 and Gln165 on the adjacent subunit (green) might be in contact with cellobiose bound at the active site of the blue subunit. The 6-methoxy group that is present in cellobiose but absent in the GX molecule is circled. Xylose is expected to bind at the reducing end site, resulting in a possible interaction with the N-terminal extension of the adjacent subunit.

By using *in vitro* competition assays, we identified xylose as a mixed inhibitor of CBP for the cellobiose phosphorolytic reaction (Figure 3A,B). The synthesis of GX from xylose and G1P, albeit slow [26], shows that xylose can bind to the CBP enzyme active site (Figure 2). This helps to explain the decrease in the apparent affinity for cellobiose in the presence of xylose (increase in K_M,app_). The decrease in maximal phosphorolytic rate of cellobiose (V_max, app_) in the presence of xylose suggests that xylose also inhibits cellobiose phosphorolytic activity in some other way, unrelated to xylose competition with cellobiose for the enzyme active site. CBP is a homodimer [27,28], and its active site pocket is formed at the interface of an (α/α)_6_-barrel domain and a helical extension from the N-terminal domain of the adjacent subunit (Figure 7) [28]. Notably, in a crystal structure of *Cellulomonas uda* CBP in complex with cellobiose [PDB: 3S4A] [29], the reducing end of the cellobiose molecule is in contact with the extension from the adjacent subunit (Figure 7). Xylose likely binds at this position, because its structure is similar to that of glucose, enabling the formation of GX from xylose and G1P. Thus, as xylose binds to and/or releases from the reducing end of the active site in one subunit, it may come into contact with the N-terminal domain of the other subunit. The interaction may alter CBP enzymatic activity, preventing cellobiose phosphorylation in the adjacent unit or resulting in decreased product dissociation from the adjacent active site.

By carrying out cellobiose and xylose co-fermentation using an efficient xylose-utilizing strain, SR8-a [20], we were able to increase the cellobiose consumption rate and decrease GX titer (Figure 4A,B), likely by keeping the steady-state intracellular xylose concentration low. The low concentrations of xylose present inside the cell would improve the apparent kinetic properties of cellobiose phosphorylation, resulting in a faster cellobiose consumption rate (Figure 3). Furthermore, with less intracellular xylose present, less substrate is available for GX formation. Thus, smaller amounts of GX are made, exported out of the cell and re-imported, reserving the capacity of the cellodextrin transporter and CBP for cellobiose phosphorolysis (Figure 6). Finally, less GX formation allows G1P to more efficiently enter into glycolysis by its conversion to G6P, thereby increasing the ethanol production rate. Thus, an efficient xylose utilization pathway can be used to alleviate cellobiose fermentation inefficiencies due to the use of CBP.

For bacteria with CBP, ORFs encoding xylose isomerase are found to co-exist, suggesting that cellobiose and xylose co-fermentation by anaerobic bacteria may be common, for example in *S. degradans*, *Cellvibrio gilvus*, *Ruminococcus sp.* and *Clostridium phytofermentans.* Although co-fermentation has not been shown with these organisms, they may have evolved means of avoiding the production of GX. Previous efforts to construct an anaerobic cellobiose and xylose co-fermentation system in *S. cerevisiae* using CBP and xylose isomerase from *Ruminococcus flavefaciens* were only partly successful [16]. This may be due to the inefficient xylose isomerase conversion step, resulting in high intracellular concentrations of xylose that negatively impact CBP-mediated cellobiose consumption.

We also explored whether CBP-mediated cellobiose conversion in the presence of xylose could be augmented by the use of a hydrolytic enzyme to cleave GX after its formation. We found that the intracellular β-glucosidase GH1-1 from *N. crassa* was capable of GX hydrolysis to glucose and xylose (Figure 5). Thus, low levels of GH1-1 co-expressed with CBP might be used to reduce GX and its associated burdens on the cellobiose consumption pathway (Figure 6). However, cellobiose was preferred as a substrate for GH1-1 in comparison to GX (Table 3) and the catalytic efficiency of GH1-1 for cellobiose is higher than that of CBP for cellobiose [10]. Hence, co-expression of GH1-1 with CBP would likely result in most of the cellobiose being hydrolyzed to glucose instead of following the phosphorolytic pathway. This effect would defeat the purpose of using CBP for its energetic advantage, because G1P generation would be replaced by glucose production. To circumvent this challenge, the intracellular β-glucosidase would need to have an increased substrate specificity for GX and lower activity for cellobiose. Protein engineering of an intracellular β-glucosidase with these properties may be feasible, because xylose is smaller than glucose. Thus, the enzyme active site of β-glucosidase could be engineered to be more bulky, allowing the binding of GX while eliminating that of cellobiose. Successes in similar protein engineering challenges have been reported [30-32].

Alternatively, CBP could be engineered to reduce or eliminate GX production. This approach is advantageous because it addresses the GX complications directly. In contrast to the use of engineered GH1-1 for GX cleavage, this approach allows the system to fully harvest the energetic advantages of the phosphorolytic pathway. However, CBP protein engineering may be challenging because xylose is a mixed-inhibitor of CBP activity (Figure 3), and therefore may require random mutagenesis, multiple site saturation mutagenesis or evolutionary engineering approaches to achieve the necessary cellobiose specificity [30,33,34].

## Conclusions

We have shown that xylose can have negative impacts on anaerobic cellobiose fermentation mediated by CBP in *S. cerevisiae*. Xylose can serve as a substrate along with G1P in a favorable reverse reaction to form the byproduct GX dimer. We have provided evidence that GX is likely exported out of cells and imported back by the exogenous cellodextrin transporter before being cleaved by SdCBP, exhausting resources that could have been reserved for cellobiose fermentation. Additionally, we identified xylose as a mixed-inhibitor of CBP activity, possibly due to the arrangement of enzyme active sites in the CBP homodimer. Cellobiose and xylose co-fermentation by the efficient xylose-utilizing SR8-a strain increased the cellobiose fermentation rate and decreased GX formation, likely by maintaining a low intracellular xylose concentration. The intracellular β-glucosidase GH1-1 from *N. crassa* was also capable of cleaving GX, and could be used to augment the CBP-mediated cellobiose consumption pathway. However, the use of an intracellular β-glucosidase alongside CBP may require further protein engineering to improve the β-glucosidase specificity for GX over cellobiose.

## Methods

### Plasmid construction

Plasmids constructed and used in this study are listed in Table 1. Plasmids containing a codon-optimized CBP gene from *S. degradans* (SdCBP) [GenBank: 90020965] [10] and cellodextrin transporter mutant from *N. crassa cdt-1* (F213L) [10] were used as templates for combining *cdt-1* (F213L) and SdCBP expression cassettes in pRS426 (pCS). SdCBP was cloned into *E. coli* expression plasmid pET302 with an N-terminal His_6_ tag to create pET-Sd. The In-Fusion HD Cloning Kit (Clontech, Mountain View, CA, USA) was used for all plasmid construction. Primers used are listed in Additional file 1: Table S1.

### Yeast strains and media

*S. cerevisiae* background strains used in this study were D452-2 (*MATα leu*2 *his*3 *ura*3 *can*1) and SR8-a (*ura*3) (Table 1). Plasmids were transformed into these strains using a standard lithium acetate yeast transformation protocol [35]. Transformants were selected on synthetic defined medium plates, which contained DOBA (MP Biomedicals, Santa Ana, CA, USA) mixed with two-fold appropriate CSM dropout mixture.

### Fermentations

Single colonies from synthetic defined plates were selected and re-streaked. Re-streaked colonies were inoculated in optimal minimal medium (oMM) supplemented with 20 g/L of cellobiose to prepare seed cultures. The oMM contained 1.7 g/L YNB Y1251 (Sigma, Saint Louis, MO, USA), two-fold appropriate CSM dropout mixture, 10 g/L (NH_4_)_2_SO_4_, 1 g/L MgSO_4_^.^7H_2_O, 6 g/L KH_2_PO_4_, 100 mg/L adenine hemisulfate, 10 mg/L inositol, 100 mg/L glutamic acid, 20 mg/L lysine, 375 mg/L serine and 0.1 M 2-(*N*-morpholino) ethanesulfonic acid (MES) pH 6.0. Seed cultures were harvested at mid- to late-exponential phase and washed twice with sterile water. Washed seed cultures were inoculated at an initial optical density at 600 nm of 20 in 200 mL serum flasks containing 50 mL of media. The flasks were closed with butyl rubber stoppers, sealed with aluminum crimps, and purged with nitrogen gas to obtain strict anaerobic fermentations. The fermentation media contained oMM supplemented with 80 g/L cellobiose, with or without 40 g/L xylose. The flasks were incubated at 30°C, 220 rpm. Extracellular concentrations of cellobiose, xylose, xylitol and ethanol were determined by high performance liquid chromatography on a Prominence HPLC (Shimadzu, Kyoto, Japan) equipped with Rezex RFQ-FastAcid H 10 × 7.8 mm column. The column was eluted with 0.01 N of H_2_SO_4_ at a flow rate of 1 mL/min, 55°C. Quantification of GX was performed using an ICS-3000 Ion Chromatography System (Dionex, Sunnyvale, CA, USA) equipped with a CarboPac® PA200 carbohydrate column. The column was eluted with a NaOAc gradient in 100 mM NaOH at a flow rate of 0.4 mL/min, 30°C.

### SdCBP protein purification

pET-Sd (pET302-NT/His_6_-SdCBP) was transformed into the BL21 (DE3) *E. coli* strain and induced by isopropyl β-D-1-thiogalactopyranoside at a final concentration of 0.2 mM. *E. coli* cells were lysed and protein purified by His^.^Bind Resin (Novagen, Darmstadt, Germany) according to supplied protocols. Purified SdCBP was stored in 20 mM MES, pH 6.0 and quantified using a NanoDrop 1000 spectrophotometer, assuming an extinction coefficient of 1.79 × 10^5^ M^-1^ cm^-1^, 280 nm.

### *In vitro* synthesis and purification of glucopyranosyl-xylose

We incubated 10 mM xylose and 10 mM G1P with and without 20 nM purified SdCBP in 20 mM MES, pH 6.0 at 37°C for 12 hours. The reaction was stopped by dilution with 0.1 M NaOH at a ratio of 1:200. Signals of components in the solutions were detected using an ICS-3000 Ion Chromatography System (Dionex) with the same conditions described above.

The synthesized GX was purified by ÄKTA Purifier (GE Healthcare Life Sciences, Munich, Germany) equipped with a Supelclean™ ENVI-Carb™ column. The column was eluted with a gradient of acetonitrile at a flow rate of 3.0 mL/min at room temperature. Purified fractions were verified using an ICS-3000 Ion Chromatography System (Dionex) with the same conditions described above.

### Mass spectrometry and tandem mass spectrometry

MS of the GX in the *in vitro* synthesis solution was performed on an LTQ XL ion trap instrument (Thermo Fisher Scientific, San Jose, CA, USA) with an electrospray ionization source operated in negative mode. The sample was introduced into the mass spectrometer by direct injection into a flow of 50% water/0.1% formic acid and 50% acetonitrile/0.1% formic acid set at a flow rate of 0.2 mL/min. The MS settings were capillary temperature 350°C, ion spray voltage 4 kV, sheath gas flow 60 (arbitrary units), auxiliary gas flow 10 (arbitrary units), sweep gas flow 5 (arbitrary units). The scan rate for full scan and MS/MS product ion scan was m/z 95 to m/z 500. The compound at m/z 357 was isolated with a m/z 2 isolation width (±1 Da) and fragmented with a normalized collision-induced dissociation energy setting of 35%. The activation time and the activation Q were 30 ms and 0.250, respectively. The mass measurement accuracy was < 3 ppm root mean square.

### Competition assay and kinetic parameters

We incubated 10 nM of purified SdCBP, 5 mM of inorganic phosphate and varying cellobiose concentrations at 30°C in 20 mM MES, pH 6.0 with 0, 2.5 or 5 mM xylose. All experiments were carried out in duplicate. G1P concentrations were detected continuously using a G1P Colorimetry Assay Kit (Sigma-Aldrich), according to the provided protocol. Initial rates at each cellobiose concentration were calculated from the rate of G1P production. Apparent kinetic parameters were determined by non-linear regression.

### *In vitro* glucopyranosyl-xylose hydrolytic activity assay

GX synthesized as described above was used at a concentration of 1 mM. The substrate was incubated with 0.5 μM of purified β-xylosidase (NCU01900) or β-glucosidase (NCU00130) in 1× PBS, pH 7.4 at 30°C for 2 hours. β-xylosidase (NCU01900) (unpublished observations by Dr. Xin Li) and β-glucosidase (NCU00130) were expressed and purified as described [6]. To stop the reactions, they were diluted with 0.1 M NaOH. Signals of components in the solutions were detected using an ICS-3000 Ion Chromatography System (Dionex) with the same conditions described above.

For kinetic parameter comparisons, 20 nM of purified GH1-1 and varying concentrations of cellobiose and purified GX were incubated at 30°C in 1× PBS, pH 7.4. All reactions were carried out in duplicate. The reactions were stopped at 0, 5, 10 and 15 minutes by addition of 0.1 M NaOH. Initial rates at each cellobiose and GX concentration were calculated from the rate of glucose production. The ICS-3000 Ion Chromatography System (Dionex) equipped with CarboPac^TM^ PA20 column was eluted with 3 mM KOH at a flow rate of 0.4 mL/min, 30°C, for glucose and xylose separation and to determine glucose concentrations in the reactions. Apparent kinetic parameters were determined by non-linear regression.

## Abbreviations

CBP: cellobiose phosphorylase; CDT-1: cellodextrin transporter from *Neurospora crassa*; CDT-1 (F213L): mutant of cellodextrin transporter from *N. crassa*; G1P: glucose-1-phosphate; G6P: glucose-6-phosphate; GH1-1: beta-glucosidase; GX: glucopyranosyl-xylose; MES: 2-(*N*-morpholino) ethanesulfonic acid; MS: mass spectrometry; MS-MS: tandem mass spectrometry; oMM: optimal minimal media; ORF: open reading frame; PBS: phosphate buffered saline; RaCBP: cellobiose phosphorylase from *Ruminococcus albus NE1*; SdCBP: cellobiose phosphorylase from *Saccharophagus degradans.*

## Competing interests

The authors declare that they have no competing interests.

## Authors’ contributions

KC and JHDC designed all experiments. KC, VK and AEG performed fermentation experiments. KC and XL prepared and purified enzymes and substrates. KC performed enzymatic assay experiments. SB performed mass spectrometry experiments. SJH, EJO, JMG and YSJ shared their preliminary observations of possible GX formation in fermentations. KC and JHDC analyzed and wrote the manuscript. All authors read and approved the manuscript.

## Supplementary Material

Additional file 1: Figure S1 Mass spectrometry of GX. Molecular mass of the synthesized dimer was quantified by MS. Expected molecular mass of GX is 312 g/mol. **(A)** Using a negative ionization mode, a 357 m/z was detected, consistent with GX plus a formate adduct. **(B)** The 357 m/z species was further analyzed by MS-MS. The 311, 179 and 131 m/z signals correspond to those expected for GX, hexose and pentose sugars, respectively. **Figure S2.** Percentage of xylose consumed accounted for by extracellular concentrations of xylitol and GX. Molar concentrations of compounds were used for the calculation. **Figure S3.** GX formation was not detected in the enzyme competition assay. Competition assay of SdCBP for cellobiose phosphorolysis was conducted in the presence of varying xylose concentrations (Figure 4). GX was not observed in any of the reactions at 15 minutes, that is, subsequent to the 0-10 minute G1P detection time points used for initial rate calculations. The signal of the product glucose overlapped with that of xylose, as indicated. The G1P signal is shown in the insert. A chromatogram of a representative reaction with 1 mM cellobiose and 5 mM xylose is shown. **Figure S4.** Fermentation profiles of engineered D452-2 and SR8-a strains in cellobiose. The strains transformed with the pCS plasmid were used in anaerobic fermentations supplied with 80 g/L of cellobiose (denoted as G2). Extracellular concentrations of **(A)** cellobiose and **(B)** ethanol are shown. **Figure S5.** Michaelis-Menten kinetic profiles of GH1-1 with **(A)** GX and **(B)** cellobiose as substrates. Kinetic parameters reported in Table 1 were calculated by non-linear curve fitting of these plots. **Table S1.** Primers used for plasmid construction. Lower case letters indicate the 15-bp overlap between fragments designed for In-Fusion cloning. Click here for file
