# Supplementary material for: Overcoming inefficient cellobiose fermentation by cellobiose phosphorylase in the presence of xylose
Source: Biotechnol Biofuels. 2014 Jun 7;7:85. doi: 10.1186/1754-6834-7-85 (PMC4061319; doi:10.1186/1754-6834-7-85)
Supplement: Additional file 1: Figure S1 — Mass spectrometry of GX. Molecular mass of the synthesized dimer was quantified by MS. Expected molecular mass of GX is 312 g/mol. (A) Using a negative ionization mode, a 357 m/z was detected, consistent with GX plus a formate adduct. (B) The 357 m/z species was further analyzed by MS-MS. The 311, 179 and 131 m/z signals correspond to those expected for GX, hexose and pentose sugars, respectively. Figure S2. Percentage of xylose consumed accounted for by extracellular concentrations of xylitol and GX. Molar concentrations of compounds were used for the calculation. Figure S3. GX formation was not detected in the enzyme competition assay. Competition assay of SdCBP for cellobiose phosphorolysis was conducted in the presence of varying xylose concentrations (Figure 4). GX was not observed in any of the reactions at 15 minutes, that is, subsequent to the 0-10 minute G1P detection time points used for initial rate calculations. The signal of the product glucose overlapped with that of xylose, as indicated. The G1P signal is shown in the insert. A chromatogram of a representative reaction with 1 mM cellobiose and 5 mM xylose is shown. Figure S4. Fermentation profiles of engineered D452-2 and SR8-a strains in cellobiose. The strains transformed with the pCS plasmid were used in anaerobic fermentations supplied with 80 g/L of cellobiose (denoted as G2). Extracellular concentrations of (A) cellobiose and (B) ethanol are shown. Figure S5. Michaelis-Menten kinetic profiles of GH1-1 with (A) GX and (B) cellobiose as substrates. Kinetic parameters reported in Table 1 were calculated by non-linear curve fitting of these plots. Table S1. Primers used for plasmid construction. Lower case letters indicate the 15-bp overlap between fragments designed for In-Fusion cloning. [file 1754-6834-7-85-S1.doc]

**Additional file 1**

**
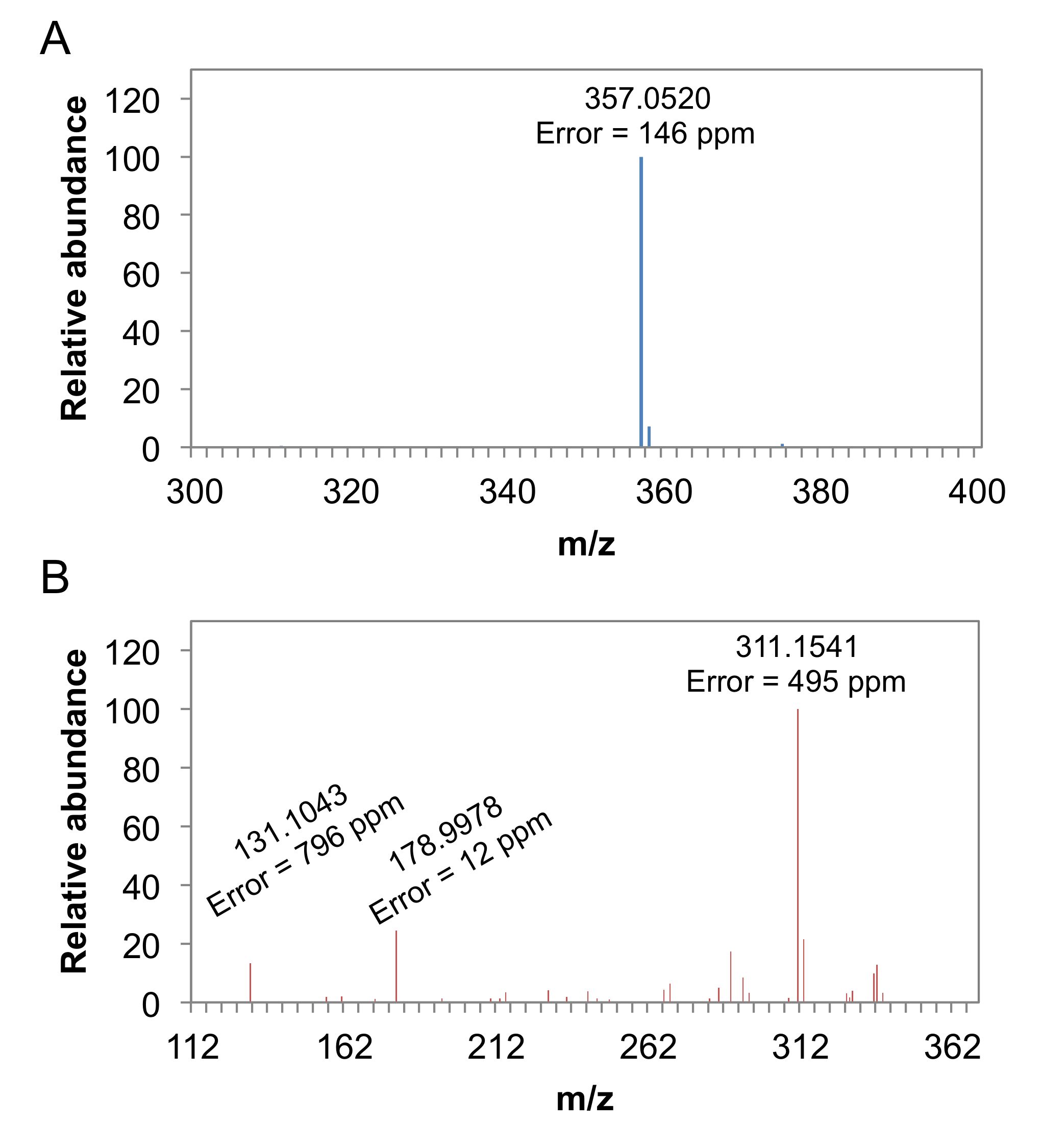
**

**Figure S1. Mass spectrometry of glucopyranosyl-xylose.** Molecular mass of the synthesized dimer was quantified using mass spectrometry (MS). The GX dimer is expected to have a molecular mass of (312 g/mol). (A) Using a negative ionization mode, a 357 m/z was detected. This is consistent with GX plus a formate adduct (312 + 46 – 1). (B) The 357 m/z species was further analyzed by tandem mass spectrometry (MS-MS). The 311, 179 and 131 m/z signals correspond to m/z ratios expected for GX, hexose and pentose sugars in the negative ionization mode, respectively.


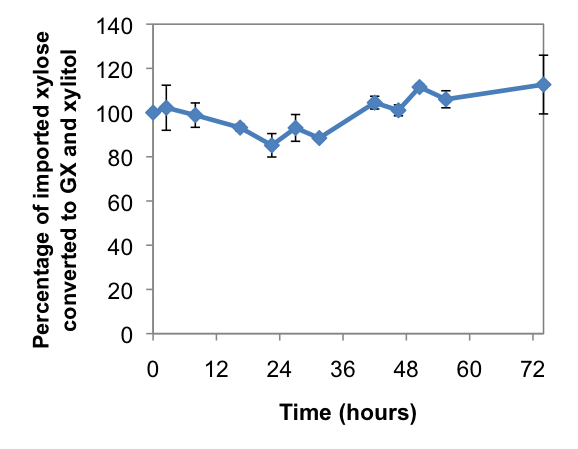


**Figure S2. Percentage of xylose consumed accounted for by extracellular concentrations of xylitol and glucopyranosyl-xylose.** The percentage was calculated by the sum of molar concentrations of xylitol and GX in the media divided by molar concentration of imported xylose (i.e. xylose no longer observable in the extracellular medium) at different time points.

**
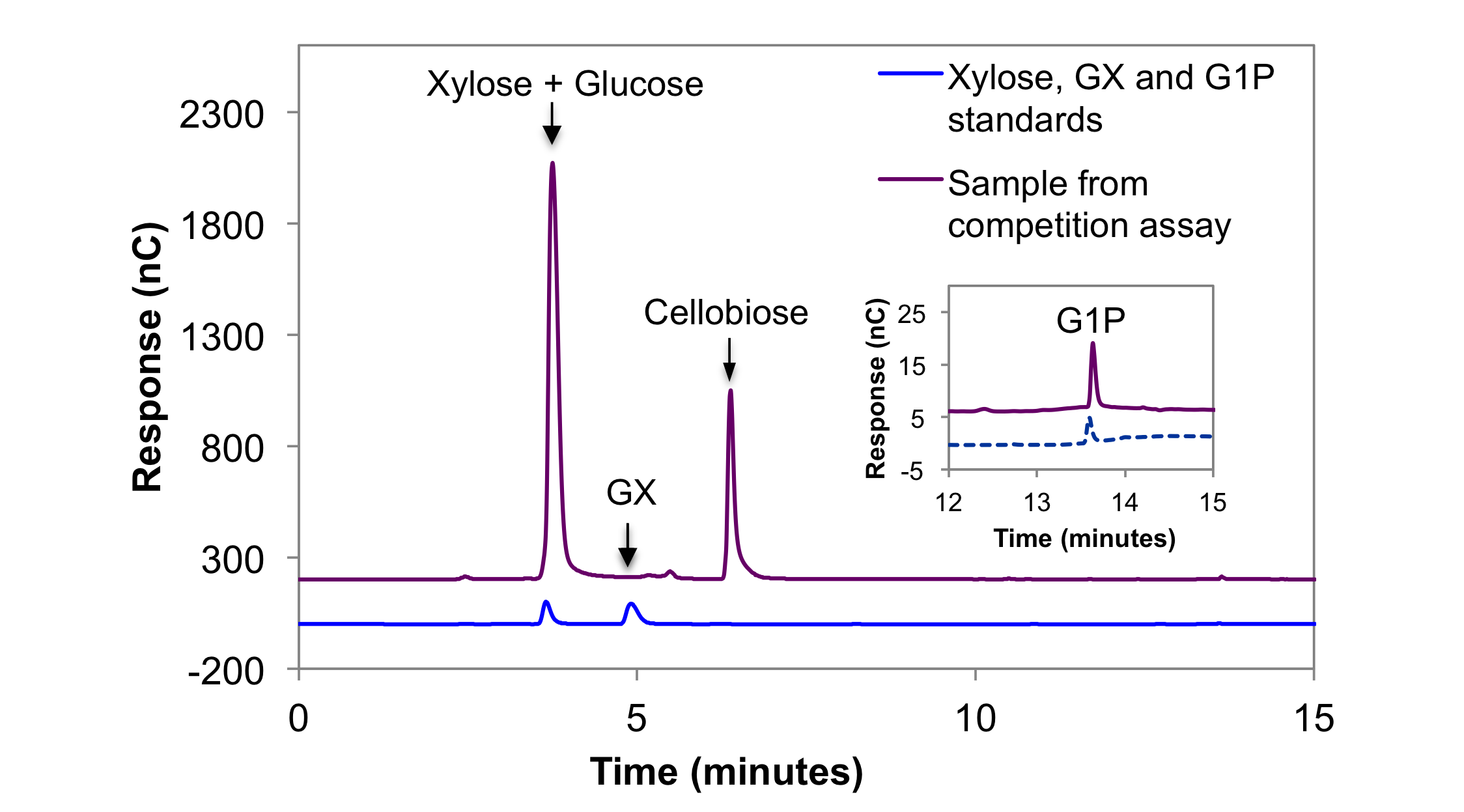
**

**Figure S3. GX formation was not detected in the enzyme competition assay.** Competition assay of SdCBP for cellobiose phosphorolysis was conducted in the presence of varying xylose concentrations (Figure 4). Concentrations of G1P, used for initial rate calculations, were detected by means of a G1P Colorimetry Assay Kit from t=0 to t=10 min. The reactions stopped by 0.1 M NaOH at t=15 minutes, i.e. subsequent to the 10-minute G1P detection time point, were analyzed by the Ion Chromatography System (Dionex). GX was not observed in any of the reactions at this time point. The signal of the product glucose overlapped with that of xylose, as indicated. The G1P signal is shown in the insert. A chromatogram of a representative reaction with 1 mM cellobiose and 5 mM xylose is shown.


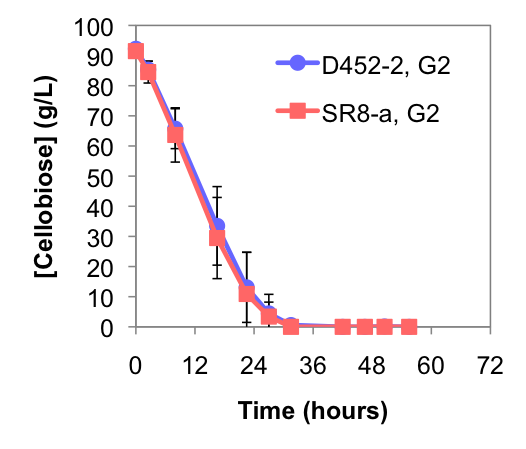

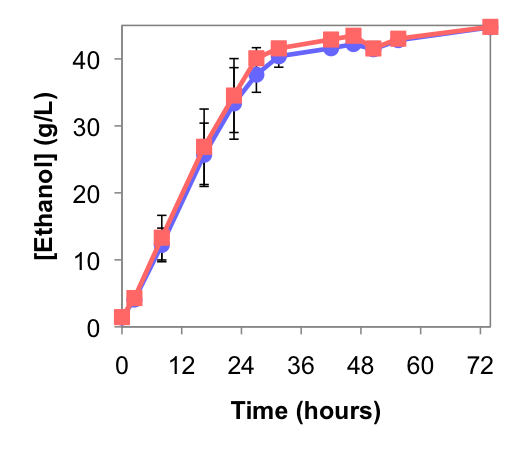


A

B

**Figure S4. Fermentation profiles of engineered D452-2 and SR8-a strains in cellobiose.** *S. cerevisiae* D452-2 (●) and SR8-a (■) strains transformed with the pCS plasmid were used in anaerobic fermentations supplied with 80 g/L of cellobiose (denoted as G2). Extracellular concentrations of cellobiose (A) and ethanol (B) are shown. Values and error bars represent the means and standard deviation of two independent biological replicates.


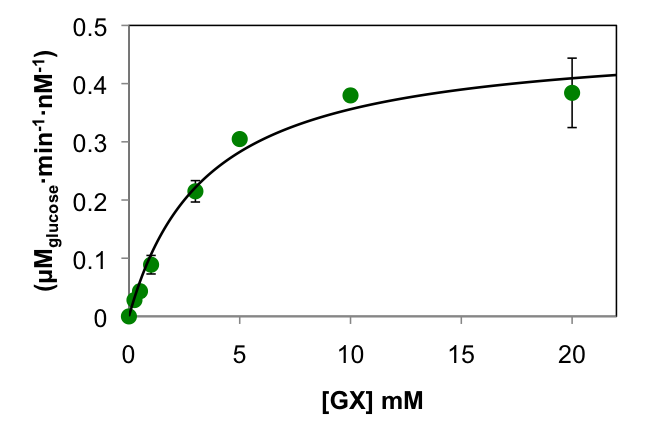


A

B


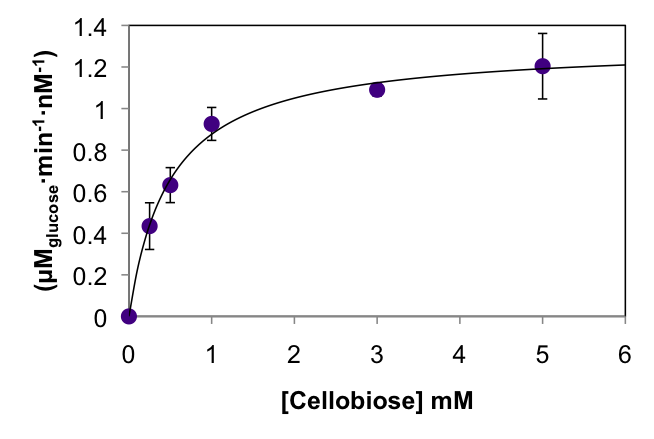


**Figure S5. Michaelis-Menten kinetic profiles of GH1-1 with glucopyranosyl-xylose and cellobiose as substrates.** (A) glucopyranosyl-xylose (●) and (B) cellobiose (●) were used as substrates for the β-glucosidase GH1-1. The initial rates were calculated from the amount of glucose released at each substrate concentration. Reactions were carried out in duplicate. Kinetic parameters reported in Table 1 were calculated by non-linear curve fitting of these plots.

**Table S1. Primers used for plasmid construction.** Lower case letters indicate the 15-bp overlap between fragments designed for In-Fusion cloning.

| **Plasmid** | **Primer Name** | **Primer sequence (5'-3')** |
| --- | --- | --- |
| **pCS** | Sd_F | tggagctcgagtgagGAACTATC |
|  | Sd_R | tactcacggccgcaaATTAAAG |
|  | cdt1_vector_F | ttgcggccgtgagtaAGGAAAG |
|  | cdt1_vector_R | ctcactcgagctccaGCTTTTGTTC |
| **pET-Sd** | pET_Sd_F | catcatcacgtgaatAAATTCGGGCACTTTG |
|  | pET_Sd_R | atccgattatacctaGCCAAGTGTTACCTCGAC |
|  | pET_vector_F | taggtataatcggatCCG |
|  | pET_vector_R | attcacgtgatgatgATG |
